# A Comparative Performance Analysis of Total PSA, Percentage Free PSA, PSA Velocity, and PSA Density versus the Detection of Primary Circulating Prostate Cells in Predicting Initial Prostate Biopsy Findings in Chilean Men

**DOI:** 10.1155/2014/676572

**Published:** 2014-07-01

**Authors:** Nigel P. Murray, Eduardo Reyes, Nelson Orellana, Cynthia Fuentealba, Ricardo Dueñas

**Affiliations:** ^1^Hospital Carabineros of Chile, Nuñoa, 7770199 Santiago, Chile; ^2^Circulating Tumor Cell Unit, Faculty of Medicine, University Mayor, Las Condes, 7550224 Santiago, Chile; ^3^Institute of Bio-Oncology, Providencia, 7500710 Santiago, Chile; ^4^Faculty of Medicine, Diego Portales University, Manuel Rodríguez Sur 415, 8370179 Santiago, Chile

## Abstract

*Introduction*. PSA parameters have been used in an attempt to improve the diagnostic yield of prostate screening tests; the detection of primary malignant circulating prostate cells (CPCs) may improve the diagnostic yield of screening and therefore avoid unnecessary biopsies. *Patients and Methods*. Prospective study of all men undergoing initial prostate biopsy due to an elevated total serum PSA. Free percent PSA, PSA velocity, and PSA density were determined. Primary CPCs were detected using standard immunocytochemistry. A positive test for CPCs was defined as one cell PSA (+) P504S (+) in an 8 ml blood sample. Positive predictive and negative predictive values, specificity, and sensitivity were calculated for each test as well as the number of biopsies avoided and cancers missed. *Results*. 303 men participated in the study of whom 113/303 (37.3%) men had prostate cancer. Of the three PSA based parameters, free percent PSA was superior, sensitivity 70.8%, and specificity 67.4%. Primary CPCs detection had a sensitivity of 88.5% and a specificity of 88.4% avoiding 181 (59.7%) biopsies, detecting 93/95 (98%) of clinically significant cancers, and missing 13 (11.5%) low grade, small volume tumors. *Conclusions*. The use of primary CPCs as a sequential test could decrease the number of initial prostate biopsies missing those cancers which are treated by active observation.

## 1. Introduction

Prostate cancer is the second most common cancer and second cause of cancer death in Chilean men [1].

Prostate specific antigen (PSA) is the only biomarker routinely used for the early detection of prostate cancer. Although PSA is highly specific for prostate, an elevated level is not specific for prostate cancer, being increased in benign pathologies [2, 3]. Consequently, approximately 70% of men with an increased serum PSA, defined as >4.0 ng/mL, do not have prostate cancer [4] and thus undergo unnecessary prostate biopsies. A PSA cutoff of 4.0 ng/mL is currently used to select men for prostate biopsy; however, this misses many cancers and it has been suggested that lowering the cutoff to 2.6 ng/mL will detect small but clinically significant cancers [5]. Attempts to use differing PSA parameters, such as age adjusted PSA, PSA density, PSA velocity, and percentage free PSA, are still controversial [6]. In this present study we addressed this important clinical situation in Chilean men presenting with an elevated total PSA or abnormal digital rectal examination and comparing these parameters with the detection of primary circulating prostate cells identified by immunocytochemistry.

## 2. Material and Methods

We prospectively studied all men undergoing an initial transrectal ultrasound guided (TRUS) prostate biopsy at the Hospital Carabineros of Chile between January 2011 and October 2012. Indications for a TRUS biopsy were an elevated total PSA, defined as >4.0 ng/mL, or a digital rectal examination (DRE) abnormal or suspicious of cancer, defined as the presence of a nodule, areas of indurations, or asymmetry in the size of the lateral lobes [7]. The data base created included age and serum PSA, and at the time of the biopsy the prostate volume was calculated using ultrasound. The pathology report of the biopsy was recorded as prostate cancer or no prostate cancer. Blood samples were taken immediately prior to prostate biopsy for the detection of primary circulating prostate cells.

*PSA Values*. Total PSA and % free PSA were measured before DRE (Siemens, Advia Centaur XR, total PSA and % free PSA); calculation of PSA velocity was performed using the log slope method, including at least three total PSA values, derived from the same assay, collected over a minimum time period of 12 months and maximum of 18 months [8]. PSA density was calculated from the ultrasound findings at the time of biopsy. Transrectal ultrasonography of the prostate was performed using an endocavitary convex probe with a 6.5 MHz transducer (Hitachi, model EVP-V33). Measures of the triaxial distances of the prostate were taken in its larger diameter and the total volume was calculated by the following formula: volume = 0.52 × transverse diameter × anteroposterior diameter × longitudinal diameter. This volume was used to calculate PSA density. Values taken to be indicative of cancer were free percentage PSA < 15 ng/mL, PSA velocity > 0.75 ng/mL/year, and PSA density > 0.15 ng/cm^3^ of prostate. All biopsies were 12-core ultrasound guided; when cancer was detected, the number of cores positive for cancer and the maximum percentage of infiltration by cancer were registered.

*Detection of Primary Circulating Prostate Cells (CPCs)*. Immediately before the biopsy, an 8 mL venous blood sample was taken in a tube containing EDTA (Beckinson-Vacutainer). Samples were maintained at 4°C and processed within 48 hours. The prostate biopsy and CPC detection were independently analyzed, with the evaluators being blinded to the clinical details and results of the biopsy or CPC test.

### 2.1. Detection of CPC

Mononuclear cells were obtained by differential centrifugation using Histopaque 1,077 (Sigma-Aldrich), washed, and resuspended in 100 *μ*L of autologous plasma. 25 *μ*L aliquots were used to make slides (silanized, DAKO, USA), dried in air for 24 hours, and fixed in a solution of 70% ethanol, 5% formaldehyde, and 25% phosphate buffered saline pH 7.4.

### 2.2. Immunocytochemistry

CPCs were detected using a monoclonal antibody directed against PSA, clone 28A4 (Novocastro Laboratory, UK), and identified using an alkaline phosphatase-anti alkaline phosphatase based system (LSAB2, DAKO, USA), with new fuchsin as the chromogen. Positive samples underwent a second process with anti-P504S clone 13H4 (DAKO, USA) and were identified with a peroxidase based system (LSAB2, DAKO, USA) with DAB (3, 3′diaminobenzidine tetrahydrochloride) as the chromogen.

A CPC was defined according to the criteria of ISHAGE (International Society of Hemotherapy and Genetic Engineering) [9] and the expression of P504S according to the Consensus of the American Association of Pathologists [10]. A malignant CPC was defined as a cell that expressed PSA and P504S, a benign prostate cell could express PSA but not P504S, and leucocytes could be P504S positive or negative but did not express PSA. A test was considered positive when at least 1 cell/4 mL of blood was detected. P504S was not used alone as leucocytes can be positive for this marker. Patients with benign CPCs were considered as being negative for the test. Prostate cancer cells as well as PIN cells express P504S whereas benign cells do not; thus cells expressing PSA and P504S were considered to be malignant, whereas cells expressing PSA but not P504S were considered to be benign [11].

### 2.3. Analysis of the Results

The discrimination of the differing diagnostic tests was defined using the normal parameters: true positive (TP); false positive (FP), false negative (FN), and true negative (TN). The predictive values, positive (PPV) as well as negative (NPV), were evaluated, as well as the positive and negative likelihood ratios (+LR and –LR, resp.). In men with FN CPC detection the details of the cancer were analyzed. The potential number of biopsies avoided for each method was calculated and the Gleason scores of missed cancers recorded. The diagnostic yield of primary CPC detection was compared with free percent PSA, PSA velocity, and PSA density. A fourth group of combined free percent PSA, PSA velocity, and PSA density was compared, whereby if one parameter of the three was positive the test was considered positive.

In addition, using the criteria of Epstein [12], the number of cancers needing active treatment and active observation were registered for each test, when the test was positive or negative, to determine the clinical significance of each test used.

### 2.4. Statistical Analysis

Descriptive statistics were used for demographic variables, expressed as mean and standard deviation in the case of continuous variables with a normal distribution. In case of an asymmetrical distribution the median and interquartile range (IQR) values were used. Noncontiguous variables were presented as frequencies. The Shapiro-Wilk test was used to determine a normal distribution. The Student *t*-Test was used to compare continuous variables with a normal distribution, the Mann-Whitney test for ordinate and continuous variables with a nonnormal distribution, and the Chi-squared test for the differences in frequency. The diagnostic yield for the test detecting CPCs and PSA-AV score were analyzed using standard parameters. For this purpose patients were classified as having or not having prostate cancer. Statistical significance was defined as a *P* value less than 0.05 to two-sided. Analysis was performed using the Stata 11.0 program (StataCorp LP, College Station, Texas, USA).

## 3. Results

303 men participated in the study, with an average age of 65.1 ± 8.9 years and a median total PSA of 5.70 ng/mL (IQR: 4.52–8.54 ng/mL). 113/303 (37.3%) men had prostate cancer diagnosed on prostate biopsy.

### 3.1. Clinical Parameters

Of the evaluated variables, % free PSA, PSA velocity, prostate volume, and PSA density were significantly different between men with positive and negative biopsy (Table 1). CPCs were detected significantly more frequently in men with a positive biopsy (*P* < 0.0001). There was no significant difference in the age of serum total PSA between men with cancer and no cancer.

### 3.2. Frequency of Cancer and No Cancer of the Differing Tests

Using the Chi-squared test the frequency of each method to detect cancer or detect no cancer was compared. The results for the differing tests are shown in Table 2.

*To Detect Cancer*. There was no significant difference comparing the use of free PSA, PSA velocity, or PSA density using the established positive cut-off values, in the frequency of detecting cancer. The frequency of cancer detection in patients with positive CPC was significantly higher than the other three tests (*P* < 0.0001).

*To Detect No Cancer*. There was no significant difference comparing the use of free PSA, PSA velocity, or PSA density using the established negative cut-off values in the frequency of detecting no cancer. The frequency of no cancer detection in men with negative CPC was significantly higher (*P* < 0.001) than the other three tests, as was that of the total versus PSA velocity (*P* = 0.02).

*Type of Cancer Detected according to the Criteria of Epstein Needing Treatment or Active Observation*. Free PSA, PSA velocity, and PSA density were not able to distinguish between cancers needing active treatment and those needing active observation. Using the combined PSA parameters or primary CPC detection, positive tests were significantly more likely to need active treatment whereas negative tests were significantly more likely to need active observation (Table 3).

In the detection of cancers needing treatment, primary CPC detection was significantly superior in comparison with the total PSA parameters (*P* = 0.003, Chi-squared test) and both were superior to single PSA parameters; there were no differences comparing single PSA parameters.

### 3.3. Diagnostic Yields

The diagnostic yields for the 4 PSA based parameters and the detection of primary CPCs are shown in Table 4.

### 3.4. Avoided Biopsies

The number of biopsies that could have been avoided by using the differing parameters and the number of missed cancers using the same criteria are shown in Table 5.

### 3.5. Gleason Score of the Cancers Not Detected by the Differing Parameters

Of the missed cancers; using free PSA 15/33 (45.5%) were Gleason 6 or above; using PSA velocity 20/46, (43.5%), PSA density 18/44 (40.9%), and CPC negative 3/13 (23.7%) respectively were Gleason 6 or more cancers. Comparing the frequency of missed cancers Gleason 4 + 5 versus Gleason ≥6 there was no significant difference between the methods (Chi-squared two-tailed test) (Table 6).

### 3.6. False Negative Results for CPC

The results of the biopsies are shown in Table 7. The majority of these cancers were low grade, small volume tumors which would be treated with active observation. Of the two Gleason 7 tumors one had one sample positive with 30% of the samples infiltrated and the other 4/12 samples positive with 15% of the samples infiltrated. The Gleason 6 tumor was an incidental finding, one sample with 2% infiltrated.

## 4. Discussion

Although total serum PSA measurement has contributed to the early detection and treatment of prostate cancer, it may be elevated in nonmalignant conditions such as benign hyperplasia and prostatitis. With a cut-off value of 4.0 ng/mL, the sensitivity has been reported as being between 67 and 80% but with a specificity of only 20–30% [13–15]. The use of PSA velocity and density and % free PSA was introduced to try to compensate for the low specificity of total PSA [16, 17], but the role of these parameters remains controversial [18, 19].

For whatever disease a screening test or program must be considered in terms of cost benefit, the benefit being the improvement in mortality and/or morbidity of the disease and the costs being the adverse effects of diagnostic procedures and treatment. In terms of benefits of prostate cancer screening, the results remain controversial. According to the UK NICE guidelines the aim of prostate biopsy is not to detect each and every prostate cancer [20]. A significant number of cancers are in men with a normal serum PSA [21]. The aim of the prostate biopsy is actually to detect those prostate cancers with the potential of causing harm.

It has been estimated that, of asymptomatic men in whom prostate cancer is detected by prostate biopsy following PSA measurement, around 50% [22] do not require active treatment. Men with clinically insignificant prostate cancers that were never destined to cause any symptoms or affect their life expectancy may not benefit from knowing that they have the “disease.” Indeed, the detection of clinically insignificant prostate cancer should be regarded as an (underrecognised) adverse effect of biopsy. The subject is further complicated by the high prevalence of prostate cancer detected at autopsy [23], the contrast between the incidence and mortality rates for prostate cancer, and the need to treat an estimated number of 37 men with screened detected prostate cancer to prevent one prostate cancer death [24, 25] and to achieve a relative mortality reduction of 40% by screening for prostate cancer [26], 50% of screened detected prostate cancers may be overtreated.

A prostate biopsy is not without adverse effects; 0.7% of men biopsied were hospitalized as a result of sepsis and/or hemorrhage [27] and thus avoiding biopsies is a worthwhile aim, if the number of clinically significant cancers detected is not prejudiced. Treatment of prostate cancer is similarly not without its adverse effects, including sexual function, urinary incontinence, and bowel problems [28, 29]. Therefore, as men with low risk or indolent prostate cancer are five times more likely to die from other diseases and the 12-year survival in this group is not improved by local therapy [30], a screening test must differentiate between these patients and those with clinical significant prostate cancer which needs treatment.

Thus an ideal biomarker for the detection of prostate cancer is one that detects clinically significant cancers, does not detect indolent cancer, and has a high negative predictive value to avoid unnecessary biopsies.

The study group represents a typical prostate biopsy population, being selected on the basis of a serum total PSA ≤ 4.0 ng/mL with 37% having prostate cancer detected. The values of free % PSA, prostate volume, and PSA density are similar to that reported in Brazilian patients [31]. PSA density at a cut-off value of >0.15 ng/mL failed to detect 44/113 (39%) of cancers, which was higher than that reported by Boulos et al. [32], and a lower sensitivity than reported by Morote et al. [33]. However, the values obtained were similar to those in Iranian men [34] and free % PSA sensitivity was similar to that reported by Morote et al. [33].

The use of PSA velocity as a screening biomarker is due to two recent developments; firstly the results from the Prostate Cancer Prevention Trial shows that there is no single cut-off value of serum PSA that separates men at high risk of prostate cancer or high grade disease from men at low risk [21]. There is a continuum of risk and the frequency of high grade disease at low PSA levels may be important. More recently NCCN guidelines [35] recommend that biopsy should be considered if total PSA is elevated or PSA velocity is >0.5 ng/mL/year. Thus the use of PSA velocity expands the definition of a positive PSA test and increases the likelihood of referral for prostate biopsy. However, the role of PSA velocity in prostate cancer detection remains controversial. In prospective screening studies PSA velocity does not appear to add value to total PSA levels and when PSA velocity values are adjusted for PSA levels it was no longer informative [21]. The Rotterdam section of the European Randomized Study of Screening for Prostate Cancer also found that PSA velocity did not improve accuracy when combined with total PSA [36]. In Chilean men PSA velocity was not more accurate than free % PSA or PSA density in terms of diagnostic yield for the detection of prostate cancer at initial biopsy.

The detection of primary CPCs had the highest diagnostic yield. It is important to emphasize that the use of primary CPC detection is as a sequential test in men with suspicion of prostate cancer and not as a primary screening test; therefore a direct comparison with performance diagnosis of the serum PSA is not possible.

What is probably more important is that the NPV of 92.8% in a sample of patients with a prevalence of cancer of 37.3% and suspicion of cancer that requires a biopsy showed that the absence of CPCs had a high discriminating power. This suggests that men with an increased serum PSA and/or abnormal DRE but negative CPC could be considered of being at low risk and thus a biopsy might not be necessary. From the point of view of the −LR of 0.13, this permits the reduction of the probability of PC in almost 40% which when applied to a prevalence of approximately 50% significantly reduces the probability of cancer posttest to around 10%. This is clinically useful when determining whether or not to continue investigating.

The test identified 98% of men with clinically significant prostate cancer and was superior to the tests using PSA parameters, alone or in combination. In prostate cancer screening program it is not routine to perform prostatic ultrasound; our data to calculate prostate volume was taken at the time of biopsy and it must be further emphasized that calculated prostate volume may differ up to 25% in comparison with the volume of radical prostatectomy specimen volume. Thus true prostate volume may differ from calculated volume, affecting PSA density values; in this study we did not use a correction factor. PSA velocity has the practical difficulty of at least 1 year of followup and may not be acceptable to patients with an increased PSA value in terms of waiting time to decide for prostate biopsy or not.

Results using the detection of circulating prostate cells and using different methodologies have been discordant. Using a dual PSA/prostate specific membrane antigen RT-PCR method Eschwège et al. [37] only found 37% of preoperative patients to be CPC positive. Davis et al. [38] found no association between CPC detection using the CellSearch system and the clinical parameters prior to radical prostatectomy or between men with local PC or controls. However, Stott et al. [39] found primary CPCs in 42% of patients with localized cancer; Fizazi et al. [40], using anti-BerEP-4 epithelial antigen combined with telomerase activity, detected primary CPCs in 79% of patients with localized cancer, a similar figure to our study. One possible reason for the wide discrepancy of results is the technology used. Regardless of the system used for isolation or enrichment, detection almost always relies on staining for cells containing cytokeratin. In those cases where EpCM has been used for cell enrichment, such as CellSearch, EpCAM can alternatively be used for detection [41]. Methods using RT-PCR have utilized anti-EpCAM or anticytokeratin based enrichment methods [41, 42]. The widely accepted concept that all positive cytokeratin and/or EpCAM and CD45 negative cells with a nucleus in cancer patients are circulating tumor cells (CTCs) has imposed a clear bias on the study of CTCs. Mainly the failure to include tumor cells that have reduced or absent cytokeratin and/or EpCAM expression and the failure to identify such cell types limit investigations into additional tumor types. EpCAM is expressed in most but not all tumors [43]; there is downregulation with cancer progression and metastasis and cytokeratins are heterogeneously expressed in tumor cells and also may be downregulated during disease progression or in poorly differentiated tumors. During the progression of epithelial to mesenchymal transition both markers are downregulated [44] and EpCAM may be downregulated to allow epithelial cell dissociation from the tumor and cytokeratin downregulated to facilitate cell plasticity and migration [45]. In this study the use of PSA and P504S to define CPCs avoids this problem, and the results are similar to that of Fizazi et al. who also avoided the use of a cytokeratin and/or EpCAM based system. The finding of CTCs that express EpCAM is not in question, but there is concern over false negatives in the failure to detect CTCs that do not express EpCAM. Using a mixture of antibodies against cell surface antigens Mikolajczyk et al. [46] showed in breast cancer patients a higher detection rate of CTCs both qualitatively and quantitatively. In breast cancer 34% of patients had EpCAM negative CTCs detected, and this difference may be one possible explanation for the difference in our findings and those of Fizazi et al. with other studies based on EpCAM and/or cytokeratins.

We believe that part of the difference documented is caused by the relatively high detection in control patients and one explication is that CPC can be found in men with prostatitis; however, these CPCs are P504S negative [47]. This underlies the problem with the different methods used to detect circulating tumor cells. This problem has been extensively reviewed as to the advantages and disadvantages of each method [48, 49]. PCR methods have a high rate of false positive results; density gradient centrifugation may be associated with increased loss of circulating cells whereas immunomagnetic separation may not recognize tumor cells which do not express EpCAM and does not differentiate between malignant and benign prostate cells.

## 5. Conclusions

In Chilean men the use of PSA density and/or PSA velocity did not improve the diagnostic yield and free serum PSA was the best of the three PSA parameters used to determine the need for a prostate biopsy in men with an elevated serum total PSA. The use of the three parameters combined improved sensitivity but at the cost of decreased specificity. In comparison the use of the detection of primary CPCs increased the diagnostic yield, decreased the number of biopsies, identified 98% of patients with clinically significant prostate cancer, and did not detect low grade small volume tumors.

## Figures and Tables

**Table 1:** Clinical variables in men with positive and negative initial biopsy.

	Positive biopsy	Negative biopsy	*P*
Age	65.5 ± 9.8	64.8 ± 8.4	*P* = 0.52
PSA ng/mL	6.10 (IQR 4.67–9.6)	5.58 (IQR 4.45–7.64)	*P* = 0.09
% free PSA	11% (IQR 9–15%)	17% (IQR 13–23)	*P* ≤ 0.001
PSA velocity ng/mL/yr	0.85 (IQR 0.59–2.05)	0.58 (IQR 0.12–1.15)	*P* = 0.006
Prostate volume	44 ± 18	57 ± 24	*P* = 0.001
PSA density	0.17 (IQR 0.11–0.25)	0.11 (IQR 0.08–0.17)	*P* = 0.006
CPC (+)	100/113	22/190	*P* < 0.0001

**Table 2:** Absolute detection rates of prostate cancer and no cancer according to test.

	Free PSA		PSA velocity		PSA density		Total		CPC		Total
	≤15%	>15%	≥0.75	<0.75	≥0.15	<0.15	(+)	(−)	(+)	(−)	
Cancer	80	33	67	46	69	44	97	16	100	13	113
No cancer	62	128	75	115	60	130	104	86	22	168	190
*P* = Chi-squared	*P* = 0.001		*P* = 0.001		*P* = 0.001		*P* < 0.0001		*P* < 0.0001		

**Table 3:** Use of the tests to define active treatment or active observation in 113 patients with prostate cancer.

Type of cancer	Free PSA		PSA velocity		PSA density		Total		CPC	
	≤15%	>15%	≥0.75	<0.75	≥0.15	<0.15	(+)	(−)	(+)	(−)
Needs treatment	71	24	58	37	61	34	82	13	93	2
Active observation	9	9	9	10	8	10	11	7	7	11
*P* = Chi-squared	*P* = 0.07		*P* = 0.25		*P* = 0.11		*P* < 0.008		*P* < 0.001	

**Table 4:** Diagnostic yields for PSA based parameters and primary CPCs.

	Sensitivity	Specificity	PPV	NPV	PLR	NLR
% free PSA ≤15%	70.8 (CI 95% 61.5–79.0)	67.4 (CI 95% 60.2–74.0)	56.3 (CI 95% 47.8–64.6)	79.5 (CI 95% 72.4–85.5)	2.17 (CI 95% 1.71–2.75)	0.43 (CI 95% 0.32–0.59)
PSA velocity >0.75 ng/mL/year	59.3 (CI 95% 49.7–68.4)	60.5 ( CI 95% 53.2–67.5)	47.2 (CI 95% 38.8–55.7)	71.4 (CI 95% 63.8–78.3)	1.50 (CI 95% 1.19–1.90)	0.67 (CI 95% 0.52–0.86)
PSA density ≥0.15	61.1 (CI 95% 51.4–70.1)	68.4 (CI 95% 61.3–75.0)	53.5 (CI 95% 44.5–62.3)	74.7 (CI 95% 67.8–81.0)	1.93 (CI 95% 1.50–2.50)	0.57 (CI 95% 0.44–0.73)
Combined	85.8 (CI 95% 78.0–91.7)	45.3 (CI 95% 38.1–52.6)	48.3 (CI 95% 41.2–55.4)	84.3 (CI 95% 75.8–90.8)	1.57 (CI 95% 1.35–1.82)	0.31 (CI 95% 0.19–0.51)
1° CPCs	88.5 (CI 95% 81.3–93.7)	88.4 (CI 95% 83.0–92.6)	82.0 (CI 95% 73.4–88.3)	92.8 (CI 95% 88.0–96.1)	7.64 (CI 95% 5.13–11.38)	0.13 (CI 95% 0.08–0.22)

PPV = positive predictive value, NPV = negative predictive value, PLR = positive likelihood ratio, and NLR = negative likelihood ratio.

**Table 5:** Number of possible avoided biopsies and missed cancers according to the parameter used to determine the need for a prostate biopsy.

	Avoided biopsies	Missed cancers (% of total cancer)	*N*° cancer/total population
% free PSA >15%	161 (53.1%)	33 (29.2%)	113/303
PSA velocity <0.75 ng/mL/year	161 (53.1%)	46 (40.7%)	113/303
PSA density <0.15 ng/mL	174 (57.%)	44 (38.9%)	113/303
CPC negative	181 (59.7%)	13 (11.5%)	113/303

**Table 6:** Gleason scores (GS) of missed cancers.

	GS4	GS5	GS6	GS7	GS8	GS9	Total
Free PSA	6	12	7	7	1	0	33
PSA velocity	8	18	8	11	1	0	46
PSA density	7	19	9	9	0	0	44
CPC negative	8	2	1	2	0	0	13

**Table 7:** Patients with false negative CPC result for prostate cancer.

	Gleason score	Number of samples positive for cancer	% of sample infiltrated
1	4	2/12	5%
2	4	2/12	5%
3	4	2/12	7%
4	4	1/12	5%
5	4	2/12	5%
6	4	1/12	5%
7	4	2/12	15%
8	4	6/12	50%
9	5	4/12	50%
10	5	2/12	3%
11	6	1/12	2%
12	7	4/12	15%
13	7	1/12	30%
